# Type 2 diabetes: a multifaceted disease

**DOI:** 10.1007/s00125-019-4909-y

**Published:** 2019-06-03

**Authors:** Ewan R. Pearson

**Affiliations:** Population Health & Genomics, School of Medicine, University of Dundee, Dundee, DD1 9SY UK

**Keywords:** Aetiology, Clustering, Complex disease, Monogenic diabetes, Palette model, Pathophysiology, Review, Type 2 diabetes

## Abstract

Type 2 diabetes is a complex disease usually diagnosed with little regard to aetiology. In the broader sense, it is a mix of different clearly defined aetiologies, such as monogenic diabetes, that we need to be better at identifying as this has major implications for treatment and patient management. Beyond this, however, type 2 diabetes is a highly heterogeneous polygenic disease. This review outlines the recent developments that recognise this heterogeneity by deconvoluting the aetiology of type 2 diabetes into pathophysiological processes, either by measuring physiological variables (such as beta cell function or insulin resistance) or using partitioned polygenic scores, and addresses recent work that clusters type 2 diabetes into distinct subgroups. Increasing evidence suggests that considering the aetiological components of type 2 diabetes matters, in terms of progression rates, treatment response and complications. In other words, clinicians need to recognise that type 2 diabetes is multifaceted and that its characteristics are important for how patients are managed.

**Electronic supplementary material:**

The online version of this article (10.1007/s00125-019-4909-y) contains a slide of the figure for download, which is available to authorised users.

## Introduction

Diabetes is simply diagnosed on the basis of hyperglycaemia, yet there are multiple complex aetiological processes that result in this diagnostic hyperglycaemia. These processes influence the phenotype of the diabetes at presentation and how the diabetes subsequently behaves in terms of progression, drug response and, probably, microvascular and macrovascular complications. The most obvious aetiological difference is the process of autoimmune destruction of beta cells, resulting in type 1 diabetes. Thus a good (but not perfect) diagnostic marker for type 1 diabetes is the presence of pancreatic autoantibodies in the blood. Other aetiological processes can be driven by single gene defects causing monogenic diabetes, such as MODY. Here again there is a (relatively) simple diagnostic test: sequencing the known monogenic diabetes genes and the identification of a pathological variant. In fact, we can dissect out the aetiology of diabetes for all instances where there is a diagnostic test (e.g. type 1 diabetes [pancreatic antibodies], haemochromatosis [ferritin, gene sequencing], Cushing’s syndrome [dexamethasone suppression test], MODY [gene sequencing], etc.) However, what is the diagnostic test for type 2 diabetes? There is none, other than by exclusion of other causes. This would suggest that the (non-)diagnostic category of type 2 diabetes is ripe for dissection by finding diagnostic biomarkers for subtypes of individuals with diabetes currently falling within this type 2 diabetes category. An alternative view is that the lack of any key diagnostic features reflects the true polygenic nature of common complex diseases and traits. We readily accept that height is polygenic and not made up of different subtypes of height (other than rare monogenic syndromes) so why is this not the case for type 2 diabetes? This review will address the complex multifaceted nature of type 2 diabetes, which in the real world reflects a mix of missed type 1 diabetes, monogenic diabetes and other diagnosable aetiologies, potentially includes a small fraction of yet to be discovered monogenic diabetes subtypes or other rare aetiologies, and includes a large group of individuals with true type 2 diabetes within whom there is considerable phenotypic variation.

## Type 2 diabetes? First exclude known aetiologies

Before we can start to consider the multiple faces of true type 2 diabetes it is important to recognise that a proportion of the individuals considered to have type 2 diabetes may have another aetiology that has not been diagnosed. There is much written on this, and this is not the focus of the review. However, it is worth making a few key points. First, an elegant paper utilising a type 1 diabetes genetic risk score in the UK Biobank cohort established that type 1 diabetes occurs at the same incidence throughout life, supporting the need to consider type 1 diabetes as an aetiology of diabetes at any age [1]. Second, there is large heterogeneity by region across the UK [2] (and the world) in genetic testing for monogenic diabetes, reflecting the difficulties of differentiating these individuals from those with type 1 or type 2 diabetes, yet we know that making a diagnosis of monogenic diabetes matters, as some patients may be able to transition off insulin treatment [3, 4]. Whilst monogenic diabetes is relatively rare, accounting for about 3% of diabetes cases diagnosed in individuals under 30 years of age, the implications for those affected are life changing. A diagnostic pipeline utilising the measurement of C-peptide and pancreatic autoantibodies to select individuals with high probability of monogenic diabetes results in improved diagnosis of monogenic diabetes and type 1 diabetes [5]. Third, there is increasing recognition of the need to get the diagnosis right at, or close to, diagnosis, and a number of studies now highlight how discriminatory the combination of clinical features, pancreatic autoantibodies and a type 1 diabetes genetic risk score are in diagnosing type 1 diabetes [6, 7]. The Exeter team have incorporated risk calculators for MODY and for type 1 diabetes within their ‘Diabetes Diagnostics’ app (via apple and android devices) or available at https://www.diabetesgenes.org/mody-probability-calculator/. With increasing application of these diagnostic biomarkers to appropriately diagnose type 1 and monogenic diabetes, we should see a reduction in the number of individuals with an incorrect label of type 2 diabetes.

## The many faces of type 2 diabetes: multiple rare subtypes?

Let’s now assume that we have excluded all individuals with a diagnosable aetiology. As outlined in the introduction, it is possible that some or all of the remaining individuals may eventually all be split into subgroups of as-yet-unknown aetiology. Over the last 10 years, genome-wide association studies (GWAS) have identified multiple common aetiological variants, each of small effect and in sum only explaining a small percentage of the heritability of diabetes. It was assumed that the missing heritability was due to multiple low-frequency and rare variants that were not being picked up in the GWAS. However, recent large-scale sequencing [8] and high-density imputation in nearly 1 million people (with and without diabetes) [9] has established that whilst rare aetiological variants can be found, they explain only a little of the phenotypic variance in type 2 diabetes, with by far the majority of the genetic variance being explained by multiple (potentially thousands) common variants. The latest study identified 80 rare variants and low-frequency variants, which in total explained 1.1% of the phenotypic variance; by contrast the 323 common variants in total explained 16.3% of the phenotypic variance [9]. Interestingly, the 2.5% of the population who are at genetically highest risk (using the top 130,000 SNPs in a polygenic risk score) have a 9.4-fold increased risk for diabetes compared with those in the lowest 2.5% of the polygenic risk score, equating to a lifetime risk of 59.7% in the highest risk group vs 6.7% in the lowest risk group in the UK population. This highlights how combining these common variants can begin to explain large differences in diabetes risk.

Whilst these large-scale population genetics studies establish that type 2 diabetes is not a composite of multiple rare subtypes, low-frequency variants of large effect have been identified and in isolated populations these can rise to high frequency and explain a large proportion of diabetes risk. In these populations it is reasonable to redefine the aetiological subtype of a group of individuals previously labelled as having type 2 diabetes on the basis of their genetic aetiology. This is beautifully highlighted in genetic studies of the Inuit population in Greenland [10]. Here, GWAS and exome sequencing identified a nonsense mutation in *TBC1D4* (also called *AS160*). This variant is present in 14% of the Inuit population, with those homozygous for the variant having a tenfold increased risk for type 2 diabetes. Overall this variant accounts for 10% of diabetes in the Inuit population (a large genetically defined subtype of diabetes) [10]. As *TBC1D4* is involved in insulin-mediated glucose uptake in muscle (via GLUT4), there is a distinct physiological phenotype among these individuals, who have marked postprandial hyperglycaemia. This raises the potential for a novel targeted treatment for this subtype of diabetes, traditionally treated according to standard type 2 diabetes guidelines.

## Heterogeneity in type 2 diabetes

**Fig. 1:**
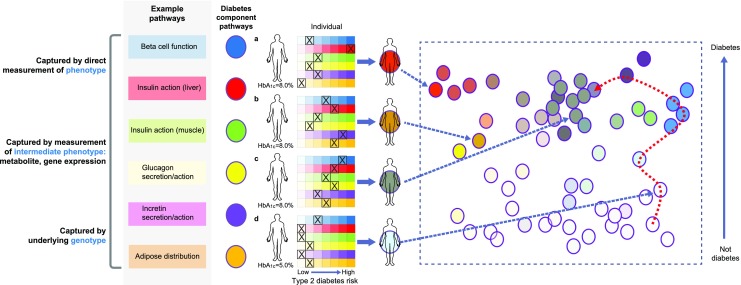
Deconvoluting the diabetes component pathways. The McCarthy palette model of diabetes represents each person as a colour, resulting from different contributions of the colours representing the various pathophysiological processes that can contribute to diabetes. It is possible to deconvolute these pathways (i.e. determine the underlying contribution made by each process) by measuring the processes directly (where possible), by measuring the underlying genetic contribution, or by measuring the intermediate metabolic phenotype (i.e. metabolites or gene expression); all three strategies could be combined in an integrative multi-omic approach. To convert values for HbA_1c_ in % into mmol/mol, subtract 2.15 and multiply by 10.929. Adapted from McCarthy [11] under the terms of the Creative Commons Attribution 4.0 International License (http://creativecommons.org/licenses/by/4.0/), which permits unrestricted use, distribution, and reproduction in any medium. This figure is available as a downloadable slide

Beyond these isolated populations, we have established that type 2 diabetes is indeed a complex polygenic disease, with limited contribution from low-frequency variants. There remains considerable variation in the phenotype of individuals with type 2 diabetes, driven of course not only by genetic variation but also by variation in lifestyle and other environmental exposures. In a recent review, McCarthy introduced the concept of the palette model of diabetes [11] in which people develop type 2 diabetes as a result of defects in multiple aetiological pathways. Many of these pathways are likely to be currently unmapped but by way of example we can label these processes: beta cell function, beta cell mass, insulin action, glucagon secretion/action, incretin secretion/action and fat distribution (Fig. 1). Each person with type 2 diabetes develops diabetes due to a combination of defects in these pathways. For many people the defect in each pathway may be subtle but, with sufficient pathways affected, diabetes results. For others, diabetes may result in a more extreme defect in one or two pathways (e.g. extreme beta cell dysfunction or severe lipodystrophy). In McCarthy’s palette model, if each pathway is given a colour, then individuals with diabetes can be represented by different shades reflecting the relative contribution of each pathophysiological process to their diabetes. Given a putative large number of pathways, most individuals would be represented by a brown colour, reflecting the small contribution made by each (or most) pathway(s). An alternative way to represent this would be if each person were plotted in a multidimensional space, with each axis reflecting the pathophysiological processes. In this context, is it possible to take a population of individuals with type 2 diabetes, map them in this space and deconvolute their aetiological processes? If so, can we use this to understand and/or predict the diabetes phenotype with respect to progression, treatment response and outcome? As outlined in Fig. 1, there are a number of routes to deconvolute the underlying aetiological mechanisms: (1) directly measure the physiological processes, where these are measurable, and along with clinical variables determine the relative contribution of each process to an individual’s phenotype; (2) measure the underlying genetic contribution, where the genetic variants are partitioned into groups reflecting the underlying aetiological process (partitioned polygenic scores); (3) measure an intermediate phenotype, such as captured by the metabolome, proteome or one of many such ‘molecular signatures’, that integrates both genetic and lifestyle factors and (4) measure and combine all of the above in an integrative multi-omic approach.

## Deconvoluting the pathways contributing to type 2 diabetes

### Clinical and physiological measures

The All New Diabetes in Scania (ANDIS) study is a large study of more than 14,000 individuals, recruited at or close to diagnosis of diabetes, with insulin, fasting glucose and antibodies measured at recruitment [12]. This unique resource has enabled a large-scale study aiming to dissect out aetiological mechanisms of all diabetes (including traditional type 1 and type 2 diabetes), with a view to grouping individuals of similar aetiology and mapping the resulting aetiological ‘subtypes’ to outcome. Ahlqvist et al. used hierarchical and *k*-means clustering of diabetes patients using age of diagnosis, HbA_1c_ at diagnosis, BMI, beta cell function (HOMA-B), insulin sensitivity (HOMA-S) and presence or absence of GAD antibodies [12]. They identified five subtypes of diabetes, which they termed severe autoimmune diabetes (SAID), otherwise known as type 1 diabetes, severe insulin-deficient diabetes (SIDD), severe insulin-resistant diabetes (SIRD), mild obesity-related diabetes (MOD) and mild age-related diabetes (MARD), and demonstrated that individuals allocated to these clusters have different characteristics. For example, the SIDD cluster progress quickly to insulin treatment and are more prone to retinopathy and the SIRD group are more prone to nephropathy. An extended critique of this analysis is beyond the scope of this review but there are few important points to address. This is an exciting study reporting, for the first time, the mapping of individuals with type 2 diabetes on a scale according to these key clinical and physiological variables at diagnosis. The fact that there is a distribution of individuals along these pathophysiological pathways is unsurprising (i.e. there are people with beta cell-deficient diabetes and people with insulin-resistant diabetes) but this study does show how type 2 diabetes varies in underlying aetiological processes (which can be determined by measuring some simple variables involved in the pathogenesis of diabetes) and how this impacts on the diabetes phenotype. However, we need to be cautious about assigning individuals to one aetiological cluster and not another. In reality there is a dense cloud of individuals in multidimensional space (or using the palette analogy, a lot are sludgy brown) that is hard to divide. *k*-means clustering forces individuals into one cluster, even if their probability of being assigned to that cluster is only marginally higher than being allocated to any of the other clusters. There is a need to establish the stability of this allocation over time and the reproducibility between populations. Furthermore, whilst clustering may be conceptually useful, it can result in loss of information when compared with using the continuous data used to undertake the clustering. Dennis et al. recently suggested that this is indeed the case [13]. These authors have undertaken clustering in the ADOPT (A Diabetes Outcome Progression Trial) clinical trial data and reproduced clusters similar to those of Ahlqvist. However, whilst they showed that the clusters are associated with differences in progression to insulin treatment, time to insulin is better predicted simply by including the age of diagnosis as a continuous measure into the model [13].

### Partitioned polygenic scores

As outlined, above, there are now known to be ~400 genetic variants associated with diabetes risk and people who carry a lot of risk variants are at marked risk of developing diabetes. These genetic variants are, by definition, aetiological variants and in the context of the palette model some people will develop diabetes as they have predominantly a genetic defect in beta cell function, whilst others will have a genetic defect in other pathways. Mahajan et al. partitioned 177 diabetes risk variants according to their association with metabolic traits, resulting in six groups of variants each characterised by a particular pathophysiological process [9]. A similar approach was undertaken by Udler et al. using 94 type 2 diabetes variants mapped to an extended range of metabolic and laboratory measures (including lipids, leptin, adiponectin) and anthropometry (including fat distribution) [14]. The resulting groups of variants (which when summed together are termed partitioned polygenic scores [pPSs]) were representative of five broad pathophysiological processes: classic beta cell deficiency with high proinsulin; beta cell deficiency with low proinsulin; obesity; lipodystrophy and a process characterised by fatty liver and abnormal lipids. Importantly, these genetically defined processes mapped well to similar physiological measures in individuals with type 2 diabetes. In addition, the resulting pPS had differential associations with outcomes (captured by population GWAS rather than in individuals with diabetes): the beta cell-deficient pPS was associated with coronary artery disease, ischaemic stroke and large and small vessel disease; the lipodystrophy pPS was associated with coronary artery disease, blood pressure and increased urinary albumin/creatinine ratio.

### Implications

With increasingly large datasets of well-characterised individuals with physiological measures and multi-omic measures, and an increasingly large number of identified diabetes risk variants, it is likely that we will be able to better map the aetiological processes that contribute to diabetes risk and map individuals within this space. For example, the IMI DIRECT (Innovative Medicines Initiative Diabetes Research on Patient Stratification) study has extensively characterised ~3000 people at different levels of blood glucose, including recently diagnosed type 2 diabetes, at baseline, at 18 months and again at 3 or 4 years [15]. These participants had frequently sampled OGTT or mixed-meal test, MRI to assess fat distribution, assessment of incretin and glucagon secretion, diet and activity assessments and multiple omics (RNA seq of blood, metabolomics [targeted and untargeted], proteomics [O-link] and faecal metagenomics). In this way a large number of potential measures including lifestyle measures can be used to tease out the aetiological processes that may contribute to diabetes risk and diabetes outcomes.

Does an understanding of the processes that contribute to type 2 diabetes and the relative contribution of these processes for an individual have any implications? Certainly, the genetic and clinical/physiological approaches have identified individuals potentially at increased risk of complications of diabetes and with different rates of progression. Thus we would anticipate that these clinical and genetic factors can be combined in risk prediction models to predict risk of diabetes progression (to failure of therapy or need for insulin) or risk of complications.

It would be interesting to see how deconvoluting the aetiological mechanisms for type 2 diabetes impacts on treatment response. Do individuals with diabetes who have a high lipodystrophy pPS have greater response to thiazolidinediones? Do those with a high beta cell deficiency pPS have altered response to sulfonylureas? This was recently demonstrated when these processes were captured by direct physiological measurement. In a clustering analysis of the ADOPT and RECORD (Rosiglitazone Evaluated for Cardiac Outcomes and Regulation of Glycaemia in Diabetes) studies, the insulin-resistant cluster responded better to thiazolidinediones and the older-patient cluster responded better to sulfonylureas [13]. However, it should be noted that, in this study, simply using age, sex, BMI and HbA_1c_ as continuous measures was much better at predicting treatment response than assigning individuals to specific clusters [13].

## Type 2 diabetes in non-white ethnic populations

This review, and indeed the bulk of the literature, largely overlooks the fact that most people with diabetes are of non-white ethnicity. What is clear is that the South Asian, East Asian and African diabetes phenotypes are markedly different from those of white populations. Whilst there are studies investigating the genetics and pathophysiology of diabetes in these populations, they are much less comprehensive than seen to date in white people. If we are truly to understand the many faces of type 2 diabetes, we need to focus on large-scale phenotyping and genotyping studies, mapped to treatment outcome and long-term outcome in these non-white populations.

## Type 2 diabetes: a multifaceted disease

Type 2 diabetes is a truly complex disease! In the broader sense, due to diagnostic challenges, it is a mix of different clearly defined aetiologies that we need to be better at identifying as this has major implications for treatment and patient management. Beyond this, type 2 diabetes is a complex disease driven by multiple pathophysiological processes resulting in a spread of clinical characteristics that to date are largely ignored when considering how we manage affected individuals. Whilst it may turn out to be clinically useful to group individuals with type 2 diabetes into subtypes based upon the main processes driving their diabetes, the case for this has not yet been made. It seems more likely that using continuous clinical and physiological measures, possibly combined with pPSs, is likely to be more valuable in predicting outcomes and guiding management.

## Electronic supplementary material

Figure slide (PPTX 279 kb)

## Abbreviations

ADOPT: A Diabetes Outcome Progression Trial
GWAS: Genome-wide association studies
pPS: Partitioned polygenic score
SIDD: Severe insulin-deficient diabetes
SIRD: Severe insulin-resistant diabetes
