# Supplementary material for: Type 2 diabetes: a multifaceted disease
Source: Diabetologia. 2019 Jun 3;62(7):1107–12. doi: 10.1007/s00125-019-4909-y (PMC6560016; doi:10.1007/s00125-019-4909-y)
Supplement: Supplementary file 1 — (PPTX 279 kb) [file 125_2019_4909_MOESM1_ESM.pptx]

## Slide 1
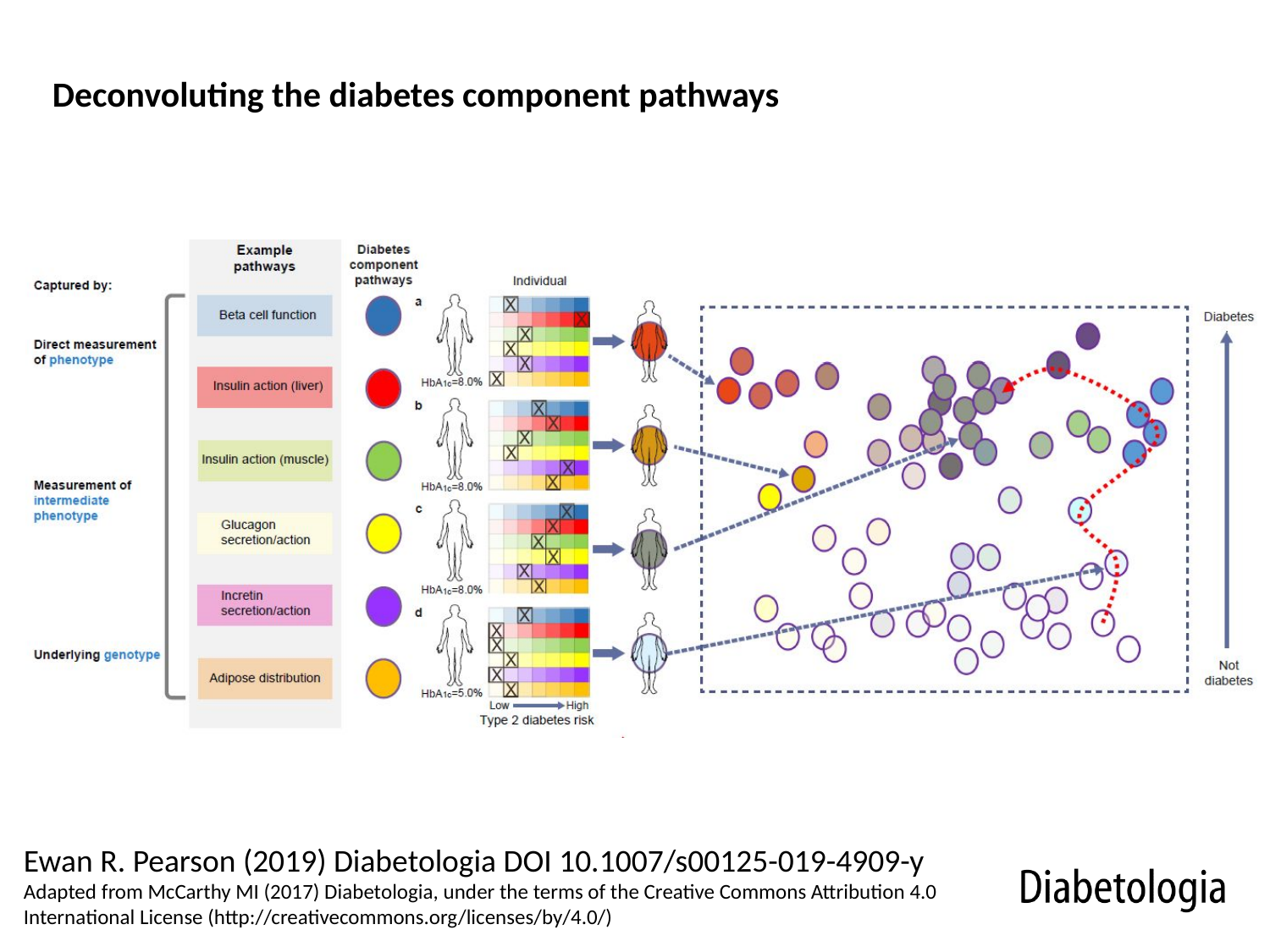

Deconvoluting the diabetes component pathways
Ewan R. Pearson (2019) Diabetologia DOI 10.1007/s00125-019-4909-y
Adapted from McCarthy MI (2017) Diabetologia, under the terms of the Creative Commons Attribution 4.0 International License (http://creativecommons.org/licenses/by/4.0/)
